# A Reliable Flow-Based Method for the Accurate Measure of Mass Density, Size and Weight of Live 3D Tumor Spheroids

**DOI:** 10.3390/mi11050465

**Published:** 2020-04-28

**Authors:** Domenico Andrea Cristaldi, Azzurra Sargenti, Simone Bonetti, Francesco Musmeci, Cecilia Delprete, Francesco Bacchi, Simone Pasqua, Carola Cavallo, Laura Bonsi, Francesco Alviano, Daniele Gazzola, Spartaco Santi

**Affiliations:** 1 Cell Dynamics isrl, via Piero Gobetti 101, 40129 Bologna, Italy; andrea.cristaldi@celldynamics.it (D.A.C.); azzurra.sargenti@celldynamics.it (A.S.); francesco.musmeci@celldynamics.it (F.M.); francesco.bacchi@celldynamics.it (F.B.); simone.pasqua@celldynamics.it (S.P.); 2 Department of Pharmacy and Biotechnology (FaBiT), Laboratory of Human and General Physiology, Università of Bologna, via San Donato 19/2, 40127 Bologna, Italy; cecilia.delprete2@unibo.it; 3 Laboratory of Preclinical Studies for Regenerative Medicine of the Musculoskeletal System (RAMSES) Laboratory, Istituto di Ricovero e Cura a Carattere Scientifico (IRCCS) Istituto Ortopedico Rizzoli, via di Barbiano 1/10, 40136 Bologna, Italy; carola.cavallo@ior.it; 4 Department of Experimental, Diagnostic and Specialty Medicine (DIMES), University of Bologna, Via Massarenti 9, 40138 Bologna, Italy; laura.bonsi@unibo.it (L.B.); francesco.alviano@unibo.it (F.A.); 5 National Research Council (CNR)-Institute of Molecular Genetics “Luigi Luca Cavalli-Sforza”, Unit of Bologna, 40136 Bologna, Italy; spartaco.santi@cnr.it; 6 IRCCS Istituto Ortopedico Rizzoli, via di Barbiano 1/10, 40136 Bologna, Italy

**Keywords:** mass density, spheroids, 3D cell culture models, multiparametric characterization, flow-reactors, analytical device

## Abstract

Gathering precise information on mass density, size and weight of cells or cell aggregates, is crucial for applications in many biomedical fields with a specific focus on cancer research. Although few technical solutions have been presented for single-cell analysis, literature does not cover this aspect for 3D models such as spheroids. Since the research interest on such samples is notably rising, here we describe a flow-apparatus, and the associated physical method and operative protocol for the accurate measurements of mass density, size and weight. The technique is based on the detection of the terminal velocity of a free-falling sample into a specifically conceived analysis flow-channel. Moreover, in order to demonstrate the accuracy and precision of the presented flow-device, analyses were initially carried out on standardized polystyrene beads. Finally, to display the application of the proposed system for biological samples, mass density, size and weight of live SW620 tumor spheroids were analyzed. The combined measurements of such parameters can represent a step toward a deeper understanding of 3D culture models.

## 1. Introduction

The past decades have been characterized by the development of three-dimensional (3D) cell culture systems, as they provide more predictive in vitro models to study fundamental cell biology, disease pathophysiology, and the identification of novel therapeutic agents [1,2,3,4,5]. 

3D culture models offer a bridge between conventional 2D in vitro testing and complex in vivo studies [6,7]. In particular, 3D cell cultures hold great potential to mimic tumors’ cellular heterogeneity, cell–cell interactions, and spatial architecture. Therefore, similarly to the in vivo analysis, these advantages allow gathering important information in fields such as drug screening, cancer research and high-throughput deep imaging [8,9]. Moreover, 3D cellular organization promotes the generation of nutrient, oxygen, signaling factor, and drug penetration, which are crucial to predict drug toxicity and/or tumor resistance [10,11,12]. 

However, the reproducibility of 3D models, in terms of size, shape and 3D architecture, can be difficult to achieve, and their generation is often time- and resource-consuming. Considering that 3D cell models assays are far less developed with respect to existing 2D methods, effort is needed for protocol improvements, as well as for the development of imaging, data acquisition, and analysis techniques [2,13,14,15]. Generally, when monitoring spheroids growth via imaging analyses, attention is focused on their size- and shape-variation over time. This gives indirect information on spheroid compactness, which is often highlighted by a transition from a loose to a tighter cell aggregate [13].

Nevertheless, this approach is not fully representative of 3D models, as spheroids undergo specific compaction processes over time, depending on factors such as cellular heterogeneity, growth environment and pharmacological treatments [16,17,18,19]. For instance, aggregates with similar dimensions can have various compositions, related to the metabolic/proliferative cell state, and the extracellular matrix deposition.

Therefore, as cell growth is characterized by variations in mass density and volume, the reliable determination of such values represents a crucial challenge to overcome [20,21]. Indeed, cells regulate more tightly density than size, and the direct measurement of cell weight and mass density can improve monitoring cell responses to external stimuli [22].

Methods for determining weight and mass density of cells, corpuscles or molecules, were established with the advent of nanoelectromechanical systems (NEMS), and in particular, with the development of nanomechanical resonators [23]. In such systems, the cantilever-like resonator oscillates at a specific frequency depending on its size and structure (i.e., stiffness). The mass density is measured by the variation in the oscillation frequency of the resonator when interacting with the sample. This technique initially needed vacuum conditions to be reliable, which represented a crucial limit to be overcome for biological applications. Suspended microchannel resonators (SMR) were therefore introduced to allow measuring the mass density of single cells, as well as deriving density distribution statistics [22,24,25].

However, the above-mentioned systems adopt microfluidic flow-channels, which make them suitable for samples up to a few micrometers in diameter. Therefore, this is a technological limitation for large biological samples, ranging from a few tens up to a few hundreds of micrometers in maximum length. 

A cost-effective alternative was presented by Belovich and Wang [26] consisting of a squared cross-sectional column for the detection of the settling velocity. The method is suitable for population analysis, but it does not allow studies on single corpuscles. Moreover, the procedure requires laborious protocols, prolonged measurements and skilled operators.

In 2012, Bach et al. calculated the mass density of polystyrene beads and phytoplankton from the measured sinking velocity [27]. This is a cost-effective solution that allows analyzing sample dimensions between 3 and 400 micrometers. Although being an efficient solution, data is collected in one step, without the possibility of repeating the measurement for a more accurate output. 

Recently, sedimentation theory, computer vision, and sample manipulation techniques were cleverly combined by Y. Zhao et al. in 2014 [28]. In this case, an optically-induced electrokinetics (OEK) system was conceived and employed for rising samples into a microfluidic channel, followed by their free-falling monitoring and consequent sedimentation velocity analysis. Similarly to the herein presented approach, polystyrene beads were used for the validation of the OEK method, and further compared with the technique developed by Bryan et al. [20]. Zhao’s solution, other than being expensive to realize, has been tested only on yeast cells, and it is not suitable for measuring samples larger than a few micrometers. Therefore, this technique does not allow reaching the average size of spheroids.

Magnetic levitation (MagLev) was also adopted for mass density measurements. Durmus et al. [29] successfully employed this technique in 2015, and the most recent application was presented by Sarigil et al. [30]. However, it can only be applied for the analysis of small samples, such as single cells.

In a recent article, J. Xie et al. [31] discussed a novel and low-cost approach in which samples up to 4 mm in size, immersed in a paramagnetic medium, can be levitated at some equilibrium position between two identical magnets. However, although a variety of samples was tested, the technique is not intended for, nor proven, for biological samples.

Therefore, here we describe a flow-apparatus, and the associated physical method and operative protocol, for the accurate and simultaneous measurement of mass density, size and weight of microbeads and sphere-like 3D models. The technique is based on the detection of the terminal velocity of a free-falling sample into a specifically conceived analysis flow-channel. Commercially available polystyrene beads were initially used for validating the method. Furthermore, in order to demonstrate the feasibility of the system for biological samples, 87 live tumor spheroids, of human colorectal carcinoma (CRC) cell line SW620, were analyzed. Notably, the obtained results demonstrated that the flow-apparatus successfully operates with samples ranging from 20 to 200 micrometers in diameter, which represent the interval most commonly used for spheroids cultured in ultra-low attachment plates.

The present device discloses, to our knowledge, the first solution to perform simultaneous or combined measurements of mass density, size and weight of cell aggregates. 

## 2. Device Architecture and the Theoretical Approach

### 2.1. Device Architecture

**Scheme 1:**
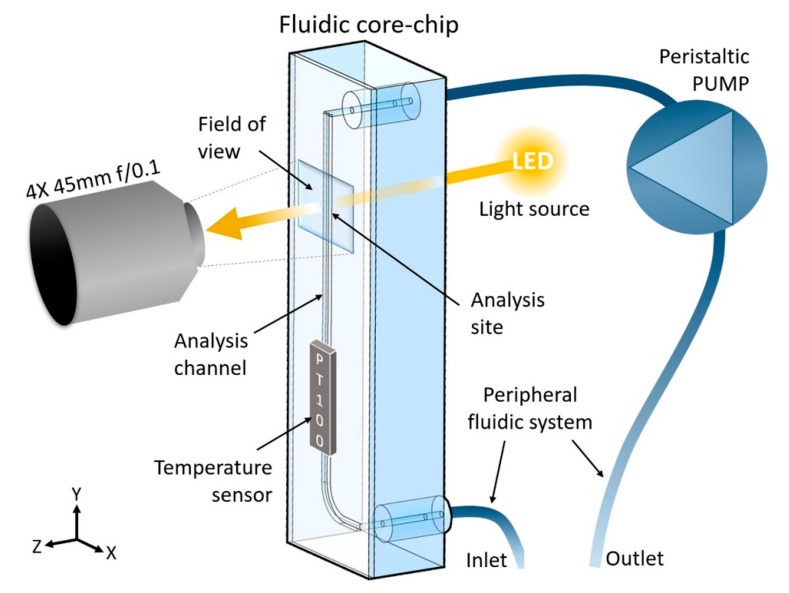
Schematic representation of the device. The fluidic core-chip is positioned vertically, whereas the light source and the optics are horizontal and at the opposite sides of the analysis site, which is the portion of the analysis channel visible through the field of view.

The device, shown in Scheme 1, comprises a fluidic core-chip that contains a specifically designed analysis channel (further described in Section 3.2), which operates in combination with a peripheral fluidic system and a peristaltic pump. Furthermore, the device is equipped with an optical system composed of a 4X objective lens (PLN 4X/0.1 Plan Achromat, FN: 22, NA: 0.1, Olympus, Tokyo, Japan), an achromatic doublet as tube lens (AC254-045-A, Thorlabs, Bergkirchen, Germany), a 13MP camera (See3CAM_CU130, e-con Systems, Karlsruhe, Germany), two aspheric lenses as collector and condenser (AL2520, Thorlabs, Bergkirchen, Germany), and a white LED SMD light source. The sample size recognition and the extrapolation of the final data are performed by the elaboration unit as further described in Section 2.2. This unit also controls the electronics, (i.e., it regulates the peristaltic pump for the introduction, distribution, analysis, and collection of samples and medium). Finally, as the temperature affects the data outputs, the device is equipped for controlling or monitoring the temperature at any time during the analysis, via a PT100 sensor positioned in proximity to the analysis channel.

### 2.2. Data Acquisition and Elaboration

**Figure 1:**
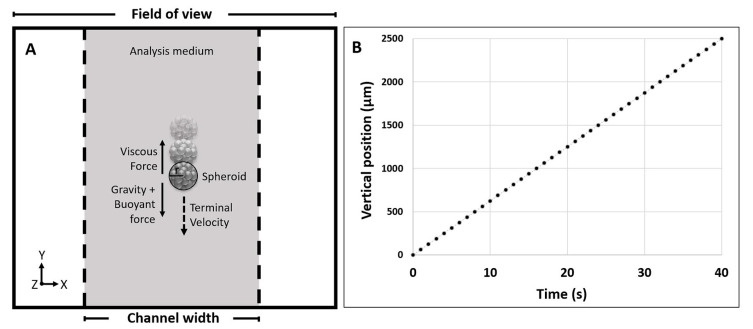
(**A**) Representation of the field of view captured by the detection unit. It shows the effect of the forces on a falling spheroid within the analysis channel; (**B**) Linear regression plot obtained for a repetition of an analyzed sample, reporting the vertical position on the Y-axis vs time (*R*^2^ = 0.9999). Differences between repetitions can be seen in Figure S1.

The output data are obtained by the combined action of the detection and the elaboration units, after calibration with a 1 mm standard reference. In wide terms, a radius is assigned to the sample that falls, driven by gravity, into the analysis site when the analysis medium is at rest. Furthermore, the motion of the sample is tracked and the described physical method (Section 2.3) is applied, taking into consideration also viscous and buoyant forces (Figure 1A). Measurements are then repeated an appropriate number of times in order to obtain statistically significant results and to maximize data reliability.

More specifically, for each brightfield image (a frame collected every 0.1 s) of the falling sample, the software assigns a circular reference by creating a threshold from the pixel grayscale. This allows extrapolating the final radius, used for the physical calculation related to the specific sample, as the average of the maximum radii obtained from each repetition.

Differently, the center of the circular reference (considered as half the diameter) is monitored for tracking the sample’ motion while free-falling. Specifically, a minimum of 10 frames, and therefore a minimum of 10 vertical positions (Y), are used to calculate the terminal velocity, and to evaluate the quality of the data by deriving a linear regression plot for each repetition (Y position vs time, Figure 1B). This allows a more accurate screening by eliminating repetitions that show an *R*^2^ value lower than 0.9999 (see Figure S1). The statistical analysis is then performed as further described in Section 3.5.

### 2.3. Physical Approach

The physical approach adopted to obtain the final output data is based on the evaluation that the free-falling sample reaches a specific constant speed, here called terminal velocity ($v_{T}$).

The $v_{T}$ is reached after a certain time in which the sample accelerates, here referred to as transient time (τ). Therefore, in order to maximize data reliability, the motion of the sample is considered only when moving at said terminal velocity, and not during the earlier transient time. The relative velocity of the sample with respect to the analysis medium *v(t)* can be obtained by solving the dynamics equation and results in the equation of motion:

(1) $$F=m_{s}a=\left(\rho_{s}-\rho_{l}\right)V_{s}g-kv$$

where *m_s_* is the mass of the sample, *ρ_s_* is the mass density of the sample, *ρ_l_* is the mass density of the liquid (referring to the analysis medium), *V_s_* is the volume of the sample derived from the measured radius (see Section 2.2), *k* is the friction coefficient, *v* is the relative velocity between the sample and the surrounding liquid, *a* is the particle acceleration, and *g* is the gravitational acceleration. Considering the definition of *k* by the Stokes law (k=6 π η r), the *v(t)* can be obtained as follow:

(2) $$v\left(t\right)=v_{T}+\left(v_{0}-v_{T}\right)\;e^{-\frac{t}{\tau }}$$

where *v_T_* is the terminal velocity, *v_0_* is the initial velocity, and *τ* is the time characteristic for the dynamics of the particle inertia. 

Said terminal velocity $v_{T}\;$and transient time τ, can finally be extrapolated as:

(3) $$v_{T}=\frac{\left(\rho_{s}-\rho_{l}\right)\;V_{s}\;g}{6\;\pi \;\eta \;r}=\frac{2}{9\eta }g\;\left(\rho_{s}-\rho_{l}\right)\;r^{2}$$

(4) $$\tau =\frac{m_{s}}{k}=\frac{\rho_{s}\;V_{s}}{6\;\pi \;\eta \;r}=\frac{2}{9\eta }\rho_{s}\;r^{2}$$

where *η* is the viscosity of the analysis medium.

Finally, the mass density $\rho_{s}\;$and weight $W_{s}$ of the sample can be obtained:

(5) $$\rho_{s}=\frac{9}{2\;g}\;\eta \;\frac{v_{T}}{r^{2}}+\rho_{l}$$

(6) $$W_{s}=\rho_{s}\;V_{s}$$

For a better understanding, here we present a calculation of the transient time τ in the case of biological samples operated in a water-based analysis medium. Specifically, knowing that the viscosity of the analysis medium depends on the temperature, the value of 1 mPas was considered, both with the mass density of the biological samples of 1020 fg/µm^3^. In the case of samples with a diameter between 1 and 2000 µm, the resulted value of τ ranges from 60 to 250 ns for the 1 and 2000 µm respectively. Therefore, it is possible to consider that, for the experimental range of the presented work (20 to 200 µm), samples reach the terminal velocity in a short amount of time, which can be considered negligible. Moreover, following the described operative method, the sample acceleration does not have an impact on the measurement, and the dynamics of the corpuscle can be safely considered as a uniform linear motion.

## 3. Materials and Methods 

### 3.1. General Procedure

In order to obtain mass density, size and weight values of the analyzed samples, the following operative protocol is needed. The inlet tube is positioned into a reservoir and the peristaltic pump is then activated to introduce the sample into the analysis channel. Specifically, the reservoir contains the analyte at a concentration sufficiently low to experimentally observe an isolated sample within the analysis site (Scheme 1). In the case of a biological sample, the operator performs the first visual screening of its quality and, by activating the flow, verifies the absence of other samples in the surrounding of the selected one, to avoid interference during measurements or repetitions. For the actual measurement, the selected sample is transported above the upper limit of the field of view, and the flow is stopped to monitor its free-falling. This is considered to be the ideal starting position in order to further minimize the transient time effect in the calculation of the terminal velocity. The operation is then repeated several times on the same sample (See Video S1). Finally, the peristaltic pump can be activated to send the sample into the waste reservoir through the outlet tubes. 

### 3.2. Fluidic Core-Chip

**Figure 2:**
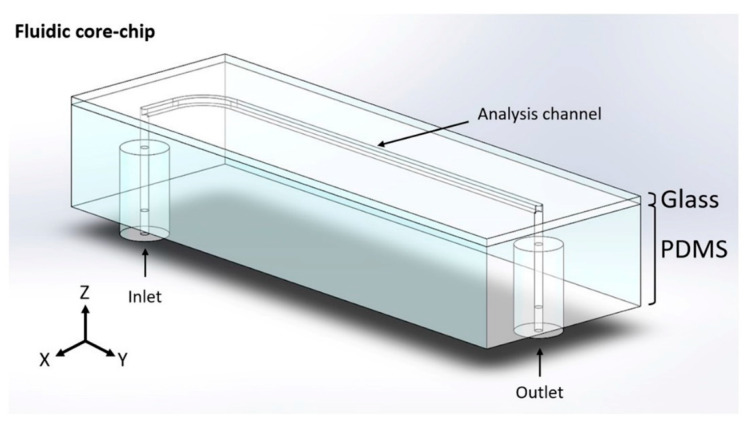
CAD design of the fluidic core-chip.

The fluidic core-chip presented in Figure 2 was made of polydimethylsiloxane (PDMS) by a stereolithographic approach and further bonded on glass via oxygen plasma treatment. More precisely, a variation of a cost-effective replica mold-casted manufacturing methods [32,33] was adopted. In particular, in order to create the positive mold, the architecture of the analysis channel was designed, using the CAD software Solid Edge^®^ (Siemens PLM Software, Plano, TX, USA), and obtained from laser-cutted aluminum by a specialized supplier (https://www.lasermio.com/). The designed piece comprises a straight portion of 60 mm in length (1.0 × 1.0 mm in cross-section) and a curved portion that connects the inlet (*r* = 5 mm). The positive mold was then created by attaching the aluminum architecture onto the bottom of a polymethylmethacrylate (PMMA) box, using a commercial epoxy glue. Moreover, two polytetrafluoroethylene (PTFE) sticks (1 mm in external diameter) were inserted into two silicone pipes (1.0 mm internal and 2.5 mm external diameter), and then connected, with a minimum amount of glue, at the initial and terminal portions of the channel. The silicone pipes will remain embedded into the PDMS chip at 3–4 mm from the channel, in order to facilitate the further connection of the fluidic core-chip to the inlet and outlet tubes. The PDMS (Sylgard 184 from Dow Corning Corporation, Midland, MI, USA) was vigorously mixed with its curing agent (10% *w*/*w*), followed by a degassing procedure under vacuum (250 millibar for 1 h). Once poured into the positive mold, the PDMS was cured in an oven at 60 °C for 4 h. Before proceeding with the peeling-off step, the PTFE sticks were extracted in order to create the inlet and outlet apertures. Finally, to obtain the final reactor, the PDMS replica architecture was bonded onto a glass slice (25 × 75 mm width × length, and 1 mm in thickness), via oxygen plasma treatment (99% air, for 60 s), using the Oxygen Plasma system Zepto (Diener, Ebhausen, Germany).

### 3.3. Validation with Polystyrene Microbeads

#### 3.3.1. Materials

In order to validate the precision and accuracy of the conceived setup, measurements were carried out on commercial polystyrene microbeads. Three batches of Polybead^®^ Polystyrene (PS) Microbeads (Polysciences Inc., Warrington, PA, USA) were purchased having the declared diameters 20, 50 and 90 μm respectively. The product sheet provided by the supplier indicates the reference mass density value of 1050 ± 10 fg/μm^3^ for the PS beads, which is considered to be equivalent to the mass density of the polystyrene chain. For the analysis medium, a Dulbecco’s phosphate buffered saline (DPBS), 1× without calcium and magnesium, pH 7.4 ± 0.1 (Corning^®^ Life Sciences, New York, NY, USA) was adopted.

#### 3.3.2. Procedure and Settings

The DPBS solution was initially flown through the entire fluidic system, until no bubbles were visible. The PS beads were then suspended in DPBS, inside a 15 mL centrifuge conical tube (Corning, New York, NY, USA) used as a reservoir, and diluted to obtain the final operating concentration of 200 beads/mL. Measurements were carried out using the previously described general procedure. For each measure, the software collected a brightfield image of the falling sample every 0.1 s. This allowed assembling at least 10 frames for the elaboration of the linear regression plot, which was then used to extrapolate the terminal velocity.

The calibration analysis was carried out under the same condition for the 20, 50 and 90 μm PS beads (in diameter). Specifically, seven different PS beads were measured for each sample size, and the mass density was calculated over seven repetitions performed for each bead. Mean and standard deviation values were calculated.

### 3.4. Spheroids Measurements

#### 3.4.1. Cell Culture and Tumor Spheroids Generation

The human colorectal carcinoma (CRC) cell line SW620 was obtained from the cell bank of the IRCCS Ospedale Policlinico San Martino, Genoa (kind gift of Dr. Alessandro Poggi MD, Head of the Molecular Oncology and Angiogenesis Unit, IRCCS Ospedale Policlinico San Martino, Genoa, Italy). SW620 cell line was cultured in RPMI 1640 (Corning, New York, NY, USA) medium supplemented with 10% fetal bovine serum (FBS, Gibco™, Thermo Fisher Scientific Italy, Monza, Italy), penicillin/streptomycin and l-glutamine (Corning, New York, NY, USA) in a humidified incubator at 37 °C with 5% CO_2_.

For the generation of tumor spheroids, SW620 adherent cells were detached with Trypsin/EDTA (Corning, New York, NY, USA) and counted using a standard hemocytometer.

Then, SW620 cell suspension was seeded at the concentration of 1.8 × 10^4^ cells per well in flat-bottom 96-well plate (Corning^®^Costar^®^, New York, NY, USA) pre-treated with Poly-HEMA 3% in EtOH 96% (P3932, Sigma Aldrich, Merck, Milano, Italy) to prevent cell adhesion, in serum-free DMEM-F12 medium (Euroclone, Milan, Italy), supplemented with epithelial growth factor (EGF) (Peprotech Europe, London, UK) at 10 ng/mL final concentration (≥1 × 10^6^ U/mg). Experiments were performed at proper spheroids compaction, after approximately 1 week of culture. 

#### 3.4.2. Procedure and Settings

For the sample preparation, the content of the 12 wells used for spheroids growth was carefully transferred, with a manual pipette, into a 15 mL centrifuge conical tube. Samples were then washed twice by centrifuging and replacing the supernatant with warm DPBS. Centrifugation was carried out at room temperature for 10 min at 800 rpm. Samples were finally diluted up to 3.5 mL into the reservoir. As previously described, samples were introduced into the device, and the operator performed the first visual screening based on samples’ morphology. Eight-seven spheroids were selected, and the mass density, size and weight were measured nine times for each sample. Only samples that showed a minimum of five repetitions with an *R*^2^ higher than 0.9999 were considered. Finally, the statistical analysis allowed the outlier elimination.

### 3.5. Statistical Analysis

The Shapiro–Wilk test was performed on the measured outputs obtained from the biological experiments in order to analyze the distribution of the data-set based on skewness and/or kurtosis [34]. 

For all the cases that resulted in a non-normal distribution, descriptive statistics box plots (Tukey method plots) were carried out for determining outliers values. This is a method for graphically depicting groups of numerical data through their quartiles. Box plots have lines extending from the boxes (whiskers) indicating variability outside the upper and lower quartiles, hence the terms box-and-whisker plot [35]. Outliers were identified as individual points for the terminal velocity, mass density, diameter, and weight box plots (see Figure S2). The presence of at least one outlier in one of the categories was considered sufficient to remove the related sample from the dataset. For these cases, the Shapiro–Wilk approach was reused to confirm the normal distribution. Data are presented as mean values ± SD.

## 4. Results and Discussion

### 4.1. Calibration Results

**Figure 3:**
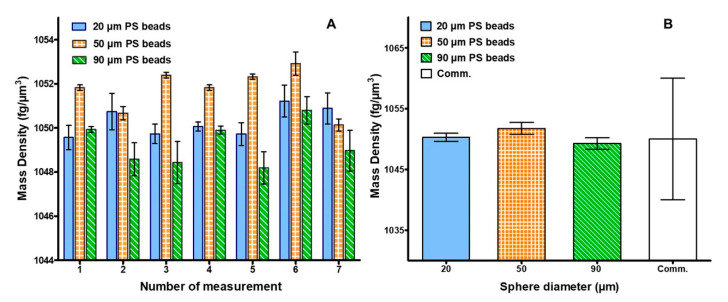
(**A**) Average mass density value and SD, over seven repetitions, for each of the seven measurements performed for the 20, 50 and 90 µm purchased PS beads (blue, orange and green respectively); (**B**) Overall average value and SD for each bead size compared to the PS assigned commercial value and SD.

The obtained mass density values are shown in Figure 3A as the average of the seven repetitions performed on each of the 21 analyzed samples (seven for each bead size). Results accuracy is highlighted by the fact that all the obtained values were included within a 5 fg/µm^3^ range. This is notable for samples averaging a mass density higher than 1000 fg/µm^3^. Specifically, minimum and maximum density values were obtained for sample 5 of the 90 µm size, and for sample 6 of the 50 µm size respectively.

Moreover, results reliability is demonstrated when comparing the average density, obtained for each bead size, to the declared commercial value of the PS beads of 1050 ± 10 fg/µm^3^ (Figure 3B). Most importantly, results derived from the described experiments were notably more accurate than the declared mass density values. In particular, Figure 3B shows that the average standard deviation for each measured bead size was one order of magnitude lower. 

A further confirmation of the accuracy of the method derived from the comparison between the theoretical calculation of the terminal velocity and the outcome obtained from the experiments. Results are shown in the supplementary materials (Figure S3), both with the determined weight of the three bead sizes.

Finally, the obtained mass densities were highly comparable with measurements previously performed on microbeads using other techniques [20,28].

### 4.2. Spheroids Measurements

After the performed measurements on microbeads, the operative size-range of the device, as well as the feasibility of the method for biological samples, were demonstrated by analyzing SW620 tumor spheroids ranging from 100 to 200 µm. The mass density, size, and weight of 87 samples were measured.

It is known that spheroids grow into irregular shapes, affecting data reliability. Therefore, in order to minimize the error due to the deviation from sphericity, the protocol presents several screening steps, summarized in (i) initial visual screening of the operator; (ii) circular reference assignment; (iii) measurements repetition; (iv) calculation of the average radius; (v) linear regression plot analysis; and (vi) statistical validation with outliers elimination.

**Figure 4:**
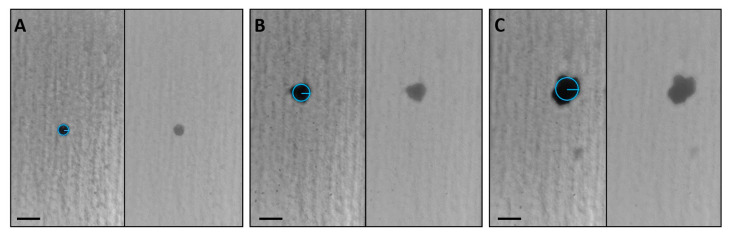
Images of frames collected for three representative samples. A small and rounded spheroid (**A**), a medium-sized sample showing a slight deviation from sphericity (**B**), and a large non-spherical outlier (**C**). For each panel, the brightfield (right) and the elaborated image (left) are shown. For better visualization, the circular references created by the software were graphically overlapped. Scale bars: 200 µm. Videos of the relative images are presented in Video S1.

Figure 4, and relative videos presented as Video S1, show three samples that represent size and shape diversity among the analyzed spheroids. Panel A displays a small sample with a well-rounded shape that matches the circular reference assigned by the software, whereas a spheroid slightly deviated from sphericity is highlighted in Panel B. Differently, the irregular sample in Panel C is an example of an outlier revealed by the statistical analysis.

**Figure 5:**
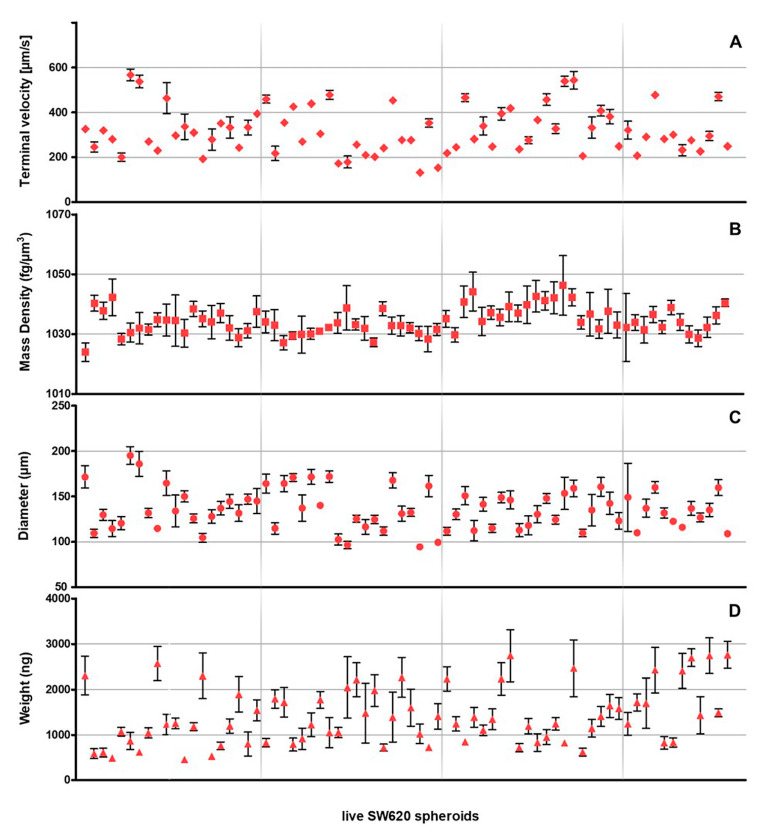
(**A**) Terminal velocity, (**B**) mass density (**C**) diameter and (**D**) weight values obtained for 72 live SW620 spheroids (rhombuses, squares, dots and triangles respectively). Each spheroid value, and the equivalent standard deviation, is extrapolated from at least five repetitions with an *R*^2^ > 0.9999.

Of the 87 measured samples, 15 were rejected by the statistical analysis, and results on the selected 72 live SW620 tumor spheroids are reported in Figure 5.

Results reported in Figure 5 show that the terminal velocity values of the analyzed SW620 spheroids variated 132 ± 12 and 570 ± 30 µm/s (Panel A), and the derived mass density values ranged between 1024 ± 3 and 1046 ± 10 fg/µm^3^ (Panel B). Moreover, sample dimensions were obtained between 95 ± 2 and 195 ± 10 µm (Panel C), while weight differences roughly reached 2300 ng, from 460 ± 20 to 2800 ± 300 ng (Panel D).

Furthermore, from Figure 5 it is possible to have a general estimation of spheroids’ sphericity, which can be related to SDs variations. It was observed that, generally, larger SDs correspond to greater irregularities. Moreover, during experiments it was also noted that, in some cases, sample irregularity caused the rotation of the spheroid while free-falling. This also contributes to the origin of the error bars, affecting the assignment of the circular reference for each frame of the specific repetition, thus influencing the elaboration of the final radius. Therefore, when samples closely resemble spheres, as shown for PS microbeads in Figure 3, SDs are smaller and display less variability.

Notably, the linear regression plot resulted in a valuable discrimination method to gather information on sample sphericity. As described in Section 3.4.2, only samples that showed at least five over nine repetitions with an *R*^2^ > 0.9999, were maintained for the final statistical analysis. Particularly, it was noted that for more spherical samples, few or no repetitions were discarded by the *R*^2^ threshold. Figure S1 shows nine linear regression plots of a representative sample, where only one repetition did not meet the required *R^2^* criteria. 

This is the first time that a quantitative measurement of mass density, and the simultaneous detection of size and weight, is performed for spheroids. 

For such samples, mass density is generally correlated with compactness, which is qualitatively measured via imaging techniques (i.e., higher cell packing densities) [36], mathematical models [37], or 3D reconstruction based on bi-dimensional images [13]. Although using different approaches and cellular models, Deisboeck et al. [37] and Sodek et al. [38] demonstrated a correlation between spheroids compactness and tumor cell invasiveness. Additionally, Murphy et al. demonstrated that mesenchymal stem cell (MSC) spheroids tend to reduce their packing density, when increasing in size, for improving the internal transport of oxygen and nutrients [39].

Other than being crucial for understanding the formation process of spheroids, mass density, size and weight are fundamental parameters for drug screening [10,13,17]. 

## 5. Conclusions

We propose a device, and relative analytical method, for the accurate, simultaneous and rapid measurement of mass density, size and weight of sphere-like samples such as microbeads, cells and spheroids. The apparatus was conceived for monitoring a sample when free-falling, driven by gravity, into a flow-channel where the analysis medium is at rest. After a first visual screening of the operator, brightfield images were acquired to assign the radius of the sample and to track its motion. After multiple repetitions on each sample, linear regression plot analyses, needed for the calculation of its terminal velocity, were performed. Moreover, the selected *R^2^* threshold of 0.9999 allowed a further screening. Finally, the proposed physical method was applied to extrapolate the output data, and results reliability was ensured by statistical analyses.

The method was first tested and validated on PS microbeads between 20 and 90 µm, and results were perfectly in line with previously presented studies. Moreover, the device was capable of measuring the mass density of PS beads with SD values 10 times lower than the commercially declared one.

Moreover, to demonstrate the feasibility of the method for biological samples, SW620 was chosen as a representative cell line for the generation of the sphere-like aggregates. Here we presented the first solution for the simultaneous measurement of mass density, size and weight of live 3D tumor spheroids by analyzing a notable number (87) of samples.

The presented results demonstrated that our method is suitable for analyzing samples ranging from 20 to 200 µm in diameter.

Further improvements can be performed based on preliminary experimental observations, specifically related to host larger spheroids, to perform specific protocols for a controlled recovery, and to evaluate samples’ sphericity. Ideally, the system, which might already host and measure samples slightly larger than 200 µm, can be easily modified to suit the desired sample dimension. This can be achieved by variating the channel size and adopting the adequate protocol based on the transient time correction. Finally, the *R*^2^ screening showed high potential as an indicator for the quantification of samples’ sphericity, deserving further attention.

Accurate mass density measurements on uni- or multicellular tumor spheroids can be precious for drug discovery and drug development fields. Information such as maturation time and compactness, can be gathered, allowing improved sample reproducibility and standardization.

## 6. Patents

A Patent Application (No. 102020000006031) incorporating parts of this work has been filed.

## Supplementary Materials

The following are available online at https://www.mdpi.com/2072-666X/11/5/465/s1, Figure S1: *R*^2^ screening; Figure S2: Tukey method plots; Figure S3: Microbeads weight and terminal velocity values; Video S1: Sample recognition example.
