# Supplementary material for: A Reliable Flow-Based Method for the Accurate Measure of Mass Density, Size and Weight of Live 3D Tumor Spheroids
Source: Micromachines (Basel). 2020 Apr 28;11(5):465. doi: 10.3390/mi11050465 (PMC7281630; doi:10.3390/mi11050465)
Supplement: Supplementary file 1 [file micromachines-11-00465-s001.pdf]

micromachines

Supplementary Materials

A Reliable Flow-Based Method for the Accurate Measure of Mass Density, Size and Weight of Live 3D Tumor Spheroids

Domenico Andrea Cristaldi [1,†], Azzurra Sargenti [1,†], Simone Bonetti [1,*], Francesco Musmeci [1], Cecilia Delprete [2], Francesco Bacchi [1], Simone Pasqua [1], Carola Cavallo [3], Laura Bonsi [4], Francesco Alviano [4], Daniele Gazzola [1,*] and Spartaco Santi [5,6]

1 Cell Dynamics isrl, via Piero Gobetti 101, 40129 Bologna, Italy; andrea.cristaldi@celldynamics.it (D.A.C.); azzurra.sargenti@celldynamics.it (A.S.); francesco.musmeci@celldynamics.it (F.M.); francesco.bacchi@celldynamics.it (F.B.); simone.pasqua@celldynamics.it (S.P.)
2 Department of Pharmacy and Biotechnology (FaBiT), Laboratory of Human and General Physiology, Università of Bologna, via San Donato 19/2, 40127 Bologna, Italy; cecilia.delprete2@unibo.it
3 Laboratory of Preclinical Studies for Regenerative Medicine of the Musculoskeletal System (RAMSES) Laboratory, Istituto di Ricovero e Cura a Carattere Scientifico (IRCCS) Istituto Ortopedico Rizzoli, via di Barbiano 1/10, 40136 Bologna, Italy; carola.cavallo@ior.it
4 Department of Experimental, Diagnostic and Specialty Medicine (DIMES), University of Bologna, Via Massarenti 9, 40138 Bologna, Italy; laura.bonsi@unibo.it (L.B.); francesco.alviano@unibo.it (F.A.)
5 National Research Council (CNR)-Institute of Molecular Genetics "Luigi Luca Cavalli-Sforza", Unit of Bologna, 40136 Bologna, Italy; spartaco.santi@cnr.it
6 IRCCS Istituto Ortopedico Rizzoli, via di Barbiano 1/10, 40136 Bologna, Italy
* Correspondence: simone.bonetti@celldynamics.it (S.B.); daniele.gazzola@celldynamics.it (D.G.)
† These authors contributed equally to this work

1. R^2 screening

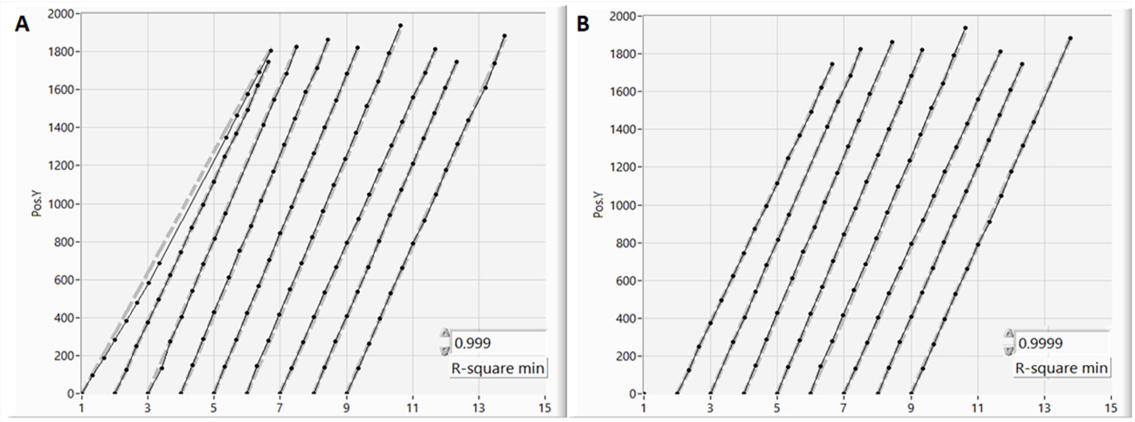

Figure S1. Panel A shows nine repetitions (X-axis) performed for a representative sample, showing that none of the linear regression plots have a lower R^2 than 0.999. However, it can be visually noted that repetition 1 presents a weaker fitting. For this reason, and due to the overall experimental observations, the R^2 threshold of 0.9999 was chosen as the standard reference for all measurements. Panel B displays the effect of the applied threshold by discarding repetition 1.

2. Tukey Method Plots

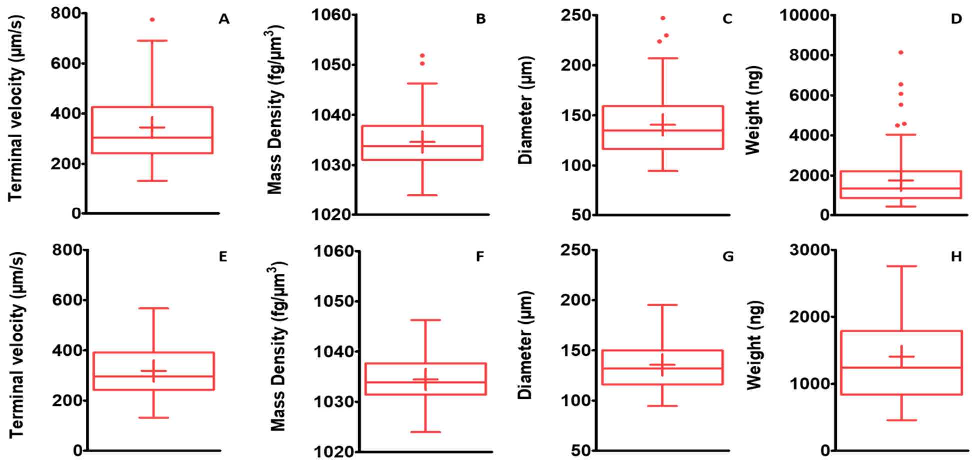

Figure S2. Tukey method plots performed for terminal velocity (**A,E**), mass density (**B,F**), diameter (**C,G**), and weight (**D,H**) values of the live SW620 spheroids analyzed. Panels from A to D (above) show the outliers identified by the statistical analysis which were then removed (Panels from E to H). The presence of at least one outlier in one of the categories was considered sufficient to remove the related sample from the dataset.

3. Microbeads Weight and Terminal Velocity Values

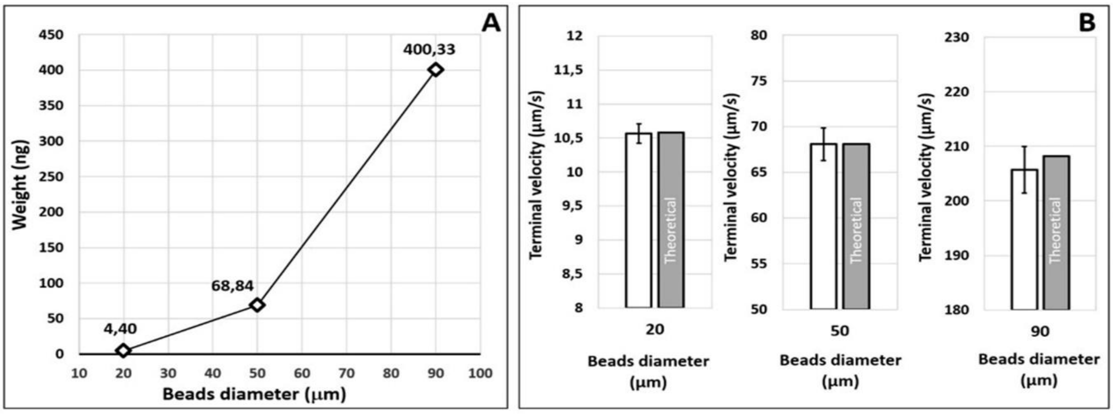

Figure S3. (**A**) Calculated weight of the 20, 50 and 90 µm PS beads; (**B**) Terminal velocity values: experimental vs theoretical results.

4. Sample Recognition Examples (Video S1)

The video shows the measurements of the three representative samples displayed in Figure 4 of the main manuscript (A, B and C). Several repetitions are shown for each sample in order to allow observing the measuring process. This included the activation of the flow system to transport the sample above the field of view for repeating the measurement. The video also demonstrates the absence of other samples during the analyses.
